# Supplementary material for: HIVprotI: an integrated web based platform for prediction and design of HIV proteins inhibitors
Source: J Cheminform. 2018 Mar 9;10:12. doi: 10.1186/s13321-018-0266-y (PMC5845081; doi:10.1186/s13321-018-0266-y)
Supplement: Supplementary file 2 — Additional file 2. Source code of HIVProtI web server. [file 13321_2018_266_MOESM2_ESM.zip › HIVprotI_Source-code/hivprotI/weka-3-6-6/doc/weka/filters/unsupervised/attribute/ClassAssigner.html]

ClassAssigner


---


|  |  |  |
| --- | --- | --- |
| **Overview** \| **Package** \| **Class** \| **Tree** \| **Deprecated** \| **Index** \| **Help** \| **Weka's home** | |  |
| **PREV CLASS**   **NEXT CLASS** | **FRAMES**    **NO FRAMES**     **All Classes** |  |
| SUMMARY: NESTED \| FIELD \| CONSTR \| METHOD | DETAIL: FIELD \| CONSTR \| METHOD |  |


---


## weka.filters.unsupervised.attribute Class ClassAssigner

```
java.lang.Object
  weka.filters.Filter
      weka.filters.SimpleFilter
          weka.filters.SimpleStreamFilter
              weka.filters.unsupervised.attribute.ClassAssigner
```

**All Implemented Interfaces:**: java.io.Serializable, CapabilitiesHandler, OptionHandler, RevisionHandler, StreamableFilter

---

``` public class ClassAssigner extends SimpleStreamFilter ```

Filter that can set and unset the class index.


Valid options are:

```
 -D
  Turns on output of debugging information.
```

```
 -C <num|first|last|0>
  The index of the class attribute. Index starts with 1, 'first'
  and 'last' are accepted, '0' unsets the class index.
  (default: last)
```

**Version:**
:   $Revision: 5543 $

**Author:**
:   fracpete (fracpete at waikato dot ac dot nz)

**See Also:**: Serialized Form

---

| **Field Summary** | |
| --- | --- |
| `static int` | `FIRST`             use the first attribute as class. |
| `static int` | `LAST`             use the last attribute as class. |
| `static int` | `UNSET`             unset the class attribute. |


**Constructor Summary**

`ClassAssigner()`


| **Method Summary** | |
| --- | --- |
| `java.lang.String` | `classIndexTipText()`             Returns the tip text for this property. |
| `Capabilities` | `getCapabilities()`             Returns the Capabilities of this filter. |
| `java.lang.String` | `getClassIndex()`             returns the class index. |
| `java.lang.String[]` | `getOptions()`             Gets the current settings of the filter. |
| `java.lang.String` | `getRevision()`             Returns the revision string. |
| `java.lang.String` | `globalInfo()`             Returns a string describing this classifier. |
| `java.util.Enumeration` | `listOptions()`             Returns an enumeration describing the available options. |
| `static void` | `main(java.lang.String[] args)`             Main method for executing this class. |
| `void` | `setClassIndex(java.lang.String value)`             sets the class index. |
| `void` | `setOptions(java.lang.String[] options)`             Parses a list of options for this object. |

**Methods inherited from class weka.filters.SimpleStreamFilter**

`batchFinished, input`

**Methods inherited from class weka.filters.SimpleFilter**

`debugTipText, getDebug, setDebug, setInputFormat`

**Methods inherited from class weka.filters.Filter**

`batchFilterFile, filterFile, getCapabilities, getOutputFormat, isFirstBatchDone, isNewBatch, isOutputFormatDefined, makeCopies, makeCopy, numPendingOutput, output, outputPeek, toString, useFilter, wekaStaticWrapper`

**Methods inherited from class java.lang.Object**

`equals, getClass, hashCode, notify, notifyAll, wait, wait, wait`

**Field Detail**

### FIRST

```
public static final int FIRST
```

:   use the first attribute as class.

    **See Also:**: Constant Field Values

---


### LAST

```
public static final int LAST
```

:   use the last attribute as class.

    **See Also:**: Constant Field Values

---


### UNSET

```
public static final int UNSET
```

:   unset the class attribute.

    **See Also:**: Constant Field Values


**Constructor Detail**

### ClassAssigner

```
public ClassAssigner()
```


**Method Detail**

### globalInfo

```
public java.lang.String globalInfo()
```

:   Returns a string describing this classifier.

    :   **Specified by:**: `globalInfo` in class `SimpleFilter`
    :   **Returns:**: a description of the classifier suitable for displaying in the explorer/experimenter gui

---


### listOptions

```
public java.util.Enumeration listOptions()
```

:   Returns an enumeration describing the available options.

    :   **Specified by:**: `listOptions` in interface `OptionHandler` **Overrides:**: `listOptions` in class `SimpleFilter`
    :   **Returns:**: an enumeration of all the available options.

---


### setOptions

```
public void setOptions(java.lang.String[] options)
                throws java.lang.Exception
```

:   Parses a list of options for this object.


    Valid options are:

    ```
     -D
      Turns on output of debugging information.
    ```

    ```
     -C <num|first|last|0>
      The index of the class attribute. Index starts with 1, 'first'
      and 'last' are accepted, '0' unsets the class index.
      (default: last)
    ```

    :   **Specified by:**: `setOptions` in interface `OptionHandler` **Overrides:**: `setOptions` in class `SimpleFilter`
    :   **Parameters:**: `options` - the list of options as an array of strings **Throws:**: `java.lang.Exception` - if an option is not supported **See Also:**: `SimpleFilter.reset()`

---


### getOptions

```
public java.lang.String[] getOptions()
```

:   Gets the current settings of the filter.

    :   **Specified by:**: `getOptions` in interface `OptionHandler` **Overrides:**: `getOptions` in class `SimpleFilter`
    :   **Returns:**: an array of strings suitable for passing to setOptions

---


### classIndexTipText

```
public java.lang.String classIndexTipText()
```

:   Returns the tip text for this property.

    :   **Returns:**: tip text for this property suitable for displaying in the explorer/experimenter gui

---


### setClassIndex

```
public void setClassIndex(java.lang.String value)
```

:   sets the class index.

    :   **Parameters:**: `value` - the class index

---


### getClassIndex

```
public java.lang.String getClassIndex()
```

:   returns the class index.

    :   **Returns:**: the class index

---


### getCapabilities

```
public Capabilities getCapabilities()
```

:   Returns the Capabilities of this filter.

    :   **Specified by:**: `getCapabilities` in interface `CapabilitiesHandler` **Overrides:**: `getCapabilities` in class `Filter`
    :   **Returns:**: the capabilities of this object **See Also:**: `Capabilities`

---


### getRevision

```
public java.lang.String getRevision()
```

:   Returns the revision string.

    :   **Specified by:**: `getRevision` in interface `RevisionHandler` **Overrides:**: `getRevision` in class `Filter`
    :   **Returns:**: the revision

---


### main

```
public static void main(java.lang.String[] args)
```

:   Main method for executing this class.

    :   **Parameters:**: `args` - should contain arguments for the filter: use -h for help


---


|  |  |  |
| --- | --- | --- |
| **Overview** \| **Package** \| **Class** \| **Tree** \| **Deprecated** \| **Index** \| **Help** \| **Weka's home** | |  |
| **PREV CLASS**   **NEXT CLASS** | **FRAMES**    **NO FRAMES**     **All Classes** |  |
| SUMMARY: NESTED \| FIELD \| CONSTR \| METHOD | DETAIL: FIELD \| CONSTR \| METHOD |  |


---
